# On the Application of a Hybrid Incomplete Exponential Sum to Aperiodic Hamming Correlation of Some Frequency-Hopping Sequences

**DOI:** 10.3390/e27090988

**Published:** 2025-09-21

**Authors:** Peihua Li, Hongyu Han

**Affiliations:** 1 Sichuan Internet College, Sichuan Normal University, Chengdu 610101, China; peihuali@sicnu.edu.cn; 2 College of Computer Science, Sichuan Normal University, Chengdu 610101, China

**Keywords:** frequency-hopping sequences, aperiodic Hamming correlation, exponential sum, hybrid sum

## Abstract

Frequency-hopping sequences are essential in frequency-hopping spread spectrum communication systems due to their strong anti-interference capabilities, low probability of interception, and high confidentiality. Existing research has predominantly focused on the periodic Hamming correlation properties of sequences, whereas the aperiodic Hamming correlation performance more accurately reflects the actual system performance. Owing to the complexity of its application scenarios and considerable research challenges, results in this area remain scarce. In this paper, we utilize exponential sums over finite fields to derive an upper bound on a hybrid incomplete exponential sum. Then, based on this upper bound, we derive bounds on the aperiodic Hamming correlation of some frequency-hopping sequence sets constructed by trace functions. Finally, by analyzing the maximum estimation error between the average and actual frequency collision numbers of such sequence sets, the validity of the derived bound is demonstrated.

## 1. Introduction

Frequency-hopping sequences (FHSs), as the core component of frequency-hopping (FH) communication systems, have been widely adopted in numerous fields such as military communications, global mobile communications, Bluetooth, HomeRF, satellite communications, underwater communications, radar, and microwave technology, due to their superior anti-interference performance and efficient multi-access networking capabilities [1,2,3,4,5]. An FH communication system typically supports multiple FH networks, each assigned an FHS as its address code. Due to differences in the start times of different networks and variations in signal transmission delays, ensuring mutual non-interference among different FHSs is challenging [6]. When two or more transmitters use the same carrier frequency to transmit signals simultaneously, their carrier frequencies might hop onto the same frequency slot, causing co-frequency collision interference. Such interference directly induces bit errors in the demodulated output at the receiver, severely degrading the quality of communication [7]. The degree of frequency coincidence between FHSs can be quantitatively characterized by the Hamming correlation function [8]. Therefore, designing FHSs with good performance has always been one of the important research topics in the study of FH communication systems.

In FH communication systems, the periodic Hamming correlation properties of FHSs determine the number of users that the system can accommodate and the error rate [9]. Over the past five decades, the academic community has established a series of classical theoretical bounds on the periodic Hamming correlation function of FHSs. Among these, the Lempel–Greenberger bound, the Peng–Fan bound, the Eun–Jin–Hong–Song bound, the Zhou–Tang–Niu–Udaya bound, and the Peng–Fan–Lee bound stand as the most representative achievements (see Refs. [10,11,12,13,14]). However, compared to the periodic Hamming correlation, the aperiodic Hamming correlation properties of FHSs can affect the performance of synchronization and sequence acquisition at a receiver [9]. From the perspective of practical applications, the aperiodic Hamming correlation function of FHSs more accurately reflects system performance. Therefore, research on the aperiodic Hamming correlation properties of FHSs possesses significant theoretical importance along with wide practical application value. However, due to the complexity of application scenarios involving aperiodic Hamming correlation, research in this domain is particularly challenging. Recently, reports on theoretical bounds on the aperiodic Hamming correlation for FHSs remain scarce (see Refs. [15,16,17,18,19]). However, to the best of our knowledge, these bounds are directly dependent on those derived from periodic Hamming correlation properties. In some cases, these derived bounds may even yield negative values, thereby rendering them meaningless for any practical analysis.

Furthermore, the hybrid exponential sum over finite fields has attracted significant attention due to its deep connections with coding theory, cryptography, and sequence design. As a core tool for investigating the number of solutions to equations over finite fields, it enables solutions to many problems intractable by other methods. However, accurately determining a hybrid exponential sum remains a highly challenging task, and current research efforts are generally limited to deriving approximate estimates [20]. Despite this, these estimates for hybrid exponential sums have been widely applied in several fields: for instance, in deriving bounds on the aperiodic inner product correlation of direct spreading sequences, establishing bounds on the performance of sequences over Galois rings, and aiding in the construction of constrained error-correcting codes and Boolean functions with high nonlinearity (see [21,22,23,24,25], and the references therein).

The purpose of this paper is twofold. Firstly, we employ exponential sums over finite fields to derive an upper bound on a hybrid incomplete exponential sum. Secondly, this upper bound is applied to derive bounds on the aperiodic Hamming correlation of some FHSs constructed via trace functions in [13]. The rest of this paper is organized as follows. In Section 2, we will provide the necessary notations and preliminary knowledge required for the following sections. In Section 3, we will present an upper bound on a hybrid incomplete exponential sum. In Section 4, we will derive and discuss the bounds on the aperiodic Hamming correlation of some FHSs. Finally, concluding remarks are given in Section 5.

## 2. Preliminaries

### 2.1. Characters of Finite Fields and Gaussian Sums

In this section, we will provide a brief introduction to the finite field theory relevant to this paper. Further details can be found in reference [26].

Let *p* be a prime number, *q* be a power of *p*, and *n* be a positive integer. For any $x\in F_{q^{n}}$, the trace function from the finite field $F_{q^{n}}$ to its subfield $F_{q}$ is defined by

$${\mathrm{Tr}}_{q^{n}/q}\left(x\right)=x+x^{q}+\cdots +x^{q^{n-1}}.$$

Let $\zeta_{p}=e^{\frac{2\pi \sqrt{-1}}{p}}$ be the *p*-th root of unity. For any $a\in F_{q^{n}}$, a nonzero function $\chi_{a}:F_{q^{n}}\to C$ is defined by

$$\chi_{a}\left(x\right)=\zeta_{p}^{Tr_{q^{n}/p}\left(ax\right)},\;\;\forall \;\;x\in F_{q^{n}}$$

and is called the additive character of $F_{q^{n}}$, where C denotes the set of complex numbers. In particular, when a=0, $\chi_{0}$ is referred to as the trivial additive character of $F_{q^{n}}$. For any $x\in F_{q^{n}}$, we have $\chi_{0}\left(x\right)=1$.

Let α be a primitive element of $F_{q^{n}}$. For each $j=0,1,\cdots ,q^{n}-2$, a nonzero function $\psi_{j}:F_{q^{n}}^{*}\to C$ is defined by

$$\psi_{j}\left(\alpha^{k}\right)=\zeta_{q^{n}-1}^{jk},\;\;\;0\leq k\leq q^{n}-2$$

and is called a multiplicative character of $F_{q^{n}}$, where $F_{q^{n}}^{*}=F_{q^{n}}\setminus \left\{0\right\}$ denotes the cyclic group of order $q^{n}-1$ and its generators are called primitive elements of $F_{q^{n}}$. In particular, when j=0, $\psi_{0}$ is referred to as the trivial multiplicative character of $F_{q^{n}}$. For any $x\in F_{q^{n}}^{*}$, we have $\psi_{0}\left(x\right)=1$.

For any character η of $F_{q^{n}}$, the character $\bar{\eta }$ defined by $\bar{\eta }\left(x\right)=\bar{\eta (x)}$ is called the conjugate character of η, where $\bar{\eta (x)}$ denotes the complex conjugate of η(x).

Let χ and ψ be an additive character and a multiplicative character of $F_{q^{n}}$, respectively. The Gaussian sum G(ψ,χ) is defined by

$$G\left(\psi ,\chi \right)=\sum_{x\in F_{q^{n}}^{*}}\psi \left(x\right)\chi \left(x\right).$$

Note that the Gaussian sum G(ψ,χ) possesses the following properties.

**Lemma 1**  ([26]). *The Gaussian sum G(ψ,χ) satisfies*

$$G\left(\psi ,\chi \right)=\left\{\begin{array}{cc} q^{n}-1, & \psi =\psi_{0},\;\chi =\chi_{0},\\ -1, & \psi =\psi_{0},\;\chi \neq \chi_{0},\\ 0, & \psi \neq \psi_{0},\;\chi =\chi_{0}. \end{array}\right.$$

*If $\psi \neq \psi_{0}$ and $\chi \neq \chi_{0}$, then*

(1) $$\begin{array}{c} \left|G\left(\psi ,\chi \right)\right|=\sqrt{q^{n}}. \end{array}$$

**Lemma 2**  ([26]). *Let χ be a non-trivial additive character of $F_{q}$ and η be a multiplicative character of $F_{q}$ of order d=gcd(n,q−1), n∈N. For any $f,h\in F_{q}$ with f≠0, then*

(2) $$\begin{array}{c} \sum_{g\in F_{q}}\chi \left(fg^{n}+h\right)=\chi \left(h\right)\sum_{t=1}^{d-1}{\bar{\eta }}^{t}\left(f\right)G\left(\eta^{t},\chi \right). \end{array}$$

In addition, let α be a primitive element of $F_{q^{n}}$, and let *e* be a positive integer such that $e|(q^{n}-1)$. For each *i* with 0≤i≤e−1, define

$$C_{i}^{\left(e,q^{n}\right)}=\alpha^{i}\langle \alpha^{e}\rangle =\left\{\alpha^{et+i}|0\leq t<\left(q^{n}-1\right)/e\right\}.$$

The cosets $C_{i}^{\left(e,q^{n}\right)}$ are called the cyclotomic classes of order *e* in $F_{q^{n}}$. If $c_{i}\in C_{i}^{\left(e,q^{n}\right)}$, then the set $\left\{c_{0},c_{1},\cdots ,c_{e-1}\right\}$ is called a complete set of representatives for the cyclotomic classes of order *e* in $F_{q^{n}}$. Obviously,

$$c_{i}C_{0}^{\left(e,q^{n}\right)}=C_{i}^{\left(e,q^{n}\right)},$$

and

$$\overset{e-1}{\underset{i=0}{⋃}}c_{i}C_{0}^{\left(e,q^{n}\right)}=F_{q^{n}}^{*}.$$

**Lemma 3**  ([27]). Let e be a positive integer such that e|(q−1) and gcd(e,n)=1. For any 0≤i≤e−1, there exists $\lambda_{i}\in F_{q}^{*}$ such that $\left\{\lambda_{0},\lambda_{1},\cdots ,\lambda_{e-1}\right\}$ is a complete set of representatives for the cyclotomic classes of order e in $F_{q^{n}}$.

### 2.2. Aperiodic Hamming Correlation Function of FHSs

Let $F=\{f_{0},f_{1},\cdots ,f_{q-1}\}$ be an alphabet of *q* available frequencies, and S be the set of all FHSs of length *N* over F. For any two frequencies $f_{i},\;f_{j}\in F$, let

$$\begin{array}{c} h\left[f_{i},f_{j}\right]=\left\{\begin{array}{cc} 1, & \;\;\mathrm{if}\;\;f_{i}=f_{j},\\ 0, & \;\;\mathrm{otherwise}. \end{array}\right. \end{array}$$

For any two FHSs $X=(x_{0},x_{1},\cdots ,x_{N-1})$ and $Y=(y_{0},y_{1},\cdots ,y_{N-1})$ in S, their aperiodic Hamming correlation function at a shift τ is defined by

$$\begin{array}{c} A_{X,Y}\left(\tau \right)=\sum_{i=0}^{N-1-\tau }h\left[x_{i},y_{i+\tau }\right],\;\;\;0\leq \tau <N, \end{array}$$

where the subscript i+τ is computed modulo *N*. In particular, when X=Y, $A_{X,X}\left(\tau \right)$ is referred to as the aperiodic Hamming auto-correlation, denoted as $A_{X}\left(\tau \right)$. When X≠Y, $A_{X,Y}\left(\tau \right)$ is referred to as the aperiodic Hamming cross-correlation. Obviously, the smaller the value of $A_{X,Y}\left(\tau \right)$, the fewer the number of collisions between the two FHSs and, thus, the lower the mutual interference.

### 2.3. Relevant Notations

For convenience, we hereby define some notations that will be used throughout the sequel.

- *q* is a power of a prime *p*;
- k,n are two positive integers with 1≤k≤n;
- *e* is a positive integer with e|(q−1) and gcd(e,n)=1;
- $\zeta_{p}=e^{\frac{2\pi \sqrt{-1}}{p}}$ is the *p*-th root of unity;
- $F_{q}$ is the finite field of order *q*;
- α is a primitive element of the finite field $F_{q^{n}}$, and $\beta =\alpha^{e}$;
- $T=\frac{q^{n}-1}{q-1}$, $N=\frac{q^{n}-1}{e}$, $d=\frac{q-1}{e}$, and $L=q^{n}-1$;
- Identifying $F_{q^{n}}$ with the *n*-dimensional $F_{q}$-vector space $F_{q}^{n}$, each element in $F_{q^{n}}$ can be viewed as a vector over $F_{q}$;
- $0_{k}=\left(0,0,\cdots ,0\right)\in F_{q}^{k}$ is a zero vector of length *k*.

## 3. An Upper Bound on a Hybrid Incomplete Exponential Sum

In this section, we will derive an upper bound on a hybrid incomplete exponential sum over finite fields, which constitutes a crucial step in determining the aperiodic Hamming correlation properties of FHSs constructed from trace functions.

**Theorem 1.** 
*Let $e_{f}\;\left(1\leq f\leq N-1\right)$ be N−1 complex numbers satisfying $\sum_{f=1}^{T-1}\left|e_{fd}\right|\leq M$, where M>0, $N=\frac{q^{n}-1}{e}$, $L=q^{n}-1$, $T=\frac{q^{n}-1}{q-1}$, and $d=\frac{q-1}{e}$. Then, for any non-trivial additive character χ of $F_{q^{n}}$, we have*

(3) $$\begin{array}{c} \left|\sum_{y\in C_{0}^{\left(e,q^{n}\right)}}\left(\sum_{f=1}^{N-1}e_{f}\sum_{x_{0}\in F_{q}^{*}}\chi \left(x_{0}y\right)\psi_{f}\left(y\right)\right)\cdots \left(\sum_{f=1}^{N-1}e_{f}\sum_{x_{k-1}\in F_{q}^{*}}\chi \left(x_{k-1}y\right)\psi_{f}\left(y\right)\right)\right|\leq \sqrt{q^{n}}{\left(Md\right)}^{k}. \end{array}$$

**Proof of Theorem 1.**  Suppose that $\hat{G}=\left\{\psi_{f}:0\leq f\leq L-1\right\}$ is the group consisting of all multiplicative characters of $F_{q^{n}}$, and $\hat{C}=\left\{\psi_{0},\psi_{N},\cdots ,\psi_{(e-1)N}\right\}$ is a subset of $\hat{G}$. Clearly, $\hat{C}$ is a subgroup of $\hat{G}$ of order *e*, and each multiplicative character $\eta \in \hat{C}$ annihilates $C_{0}^{\left(e,q^{n}\right)}$, i.e., η(y)=1 for all $y\in C_{0}^{\left(e,q^{n}\right)}$. Then, we have

$$\begin{array}{c} \sum_{\eta \in \hat{C}}\eta \left(y\right)=\left\{\begin{array}{cc} e, & \mathrm{if}\;\;y\in C_{0}^{\left(e,q^{n}\right)},\\ 0, & \mathrm{otherwise}, \end{array}\right. \end{array}$$

and

$$\begin{array}{cc} \; & \left|\sum_{y\in C_{0}^{\left(e,q^{n}\right)}}\left(\sum_{x_{0}\in F_{q}^{*}}\chi \left(x_{0}y\right)\psi_{f}\left(y\right)\right)\cdots \left(\sum_{x_{k-1}\in F_{q}^{*}}\chi \left(x_{k-1}y\right)\psi_{f}\left(y\right)\right)\right|\\ = & \left|e^{-k}\sum_{y\in F_{q^{n}}^{*}}\left(\sum_{x_{0}\in F_{q}^{*}}\chi \left(x_{0}y\right)\psi_{f}\left(y\right)\sum_{\eta \in \hat{C}}\eta \left(y\right)\right)\cdots \left(\sum_{x_{k-1}\in F_{q}^{*}}\chi \left(x_{k-1}y\right)\psi_{f}\left(y\right)\sum_{\eta \in \hat{C}}\eta \left(y\right)\right)\right|\\ = & \left|e^{-k}\sum_{y\in F_{q^{n}}^{*}}\left(\sum_{x_{0}\in F_{q}^{*}}\sum_{\eta \in \hat{C}}\chi \left(x_{0}y\right)\psi_{f}\left(y\right)\eta \left(y\right)\right)\cdots \left(\sum_{x_{k-1}\in F_{q}^{*}}\sum_{\eta \in \hat{C}}\chi \left(x_{k-1}y\right)\psi_{f}\left(y\right)\eta \left(y\right)\right)\right|\\ = & \left|e^{-k}\sum_{y\in F_{q^{n}}^{*}}\left(\sum_{x_{0}\in F_{q}^{*}}\sum_{\eta \in \hat{C}}\chi \left(x_{0}y\right)\psi_{f}\left(y\right)\eta \left(y\right)\psi_{f}\left(x_{0}\right)\eta \left(x_{0}\right){\bar{\psi }}_{f}\left(x_{0}\right)\bar{\eta }\left(x_{0}\right)\right)\cdots \right.\\ \; & \;\;\;\;\;\;\;\;\;\;\;\;\;\;\;\;\left.\left(\sum_{x_{k-1}\in F_{q}^{*}}\sum_{\eta \in \hat{C}}\chi \left(x_{k-1}y\right)\psi_{f}\left(y\right)\eta \left(y\right)\psi_{f}\left(x_{k-1}\right)\eta \left(x_{k-1}\right){\bar{\psi }}_{f}\left(x_{k-1}\right)\bar{\eta }\left(x_{k-1}\right)\right)\right|\\ = & \left|e^{-k}\sum_{y\in F_{q^{n}}^{*}}\left(\sum_{x_{0}\in F_{q}^{*}}\sum_{\eta \in \hat{C}}\chi \left(x_{0}y\right)\psi_{f}\left(x_{0}y\right)\eta \left(x_{0}y\right){\bar{\psi }}_{f}\left(x_{0}\right)\bar{\eta }\left(x_{0}\right)\right)\cdots \right.\\ \; & \;\;\;\;\;\;\;\;\;\;\;\;\;\;\;\;\;\left.\left(\sum_{x_{k-1}\in F_{q}^{*}}\sum_{\eta \in \hat{C}}\chi \left(x_{k-1}y\right)\psi_{f}\left(x_{k-1}y\right)\eta \left(x_{k-1}y\right){\bar{\psi }}_{f}\left(x_{k-1}\right)\bar{\eta }\left(x_{k-1}\right)\right)\right|. \end{array}$$

Let $\kappa \left(xy\right)=\psi_{f}\left(xy\right)\eta \left(xy\right)$. For any 0≤j≤e−1 and 1≤f≤N−1, since f+jN≠0(modL) and $\eta \in \hat{C}$, it follows that $\psi_{f}\eta \neq \psi_{0}$. Thus, κ is a non-trivial multiplicative character of $F_{q^{n}}$. For any $x\in F_{q}^{*}$, according to Equation (1) in Lemma 1, we have

$$\begin{array}{c} \left|\sum_{y\in F_{q^{n}}^{*}}\chi \left(xy\right)\kappa \left(xy\right)\right|=\left|\sum_{y\in F_{q^{n}}^{*}}\chi \left(y\right)\kappa \left(y\right)\right|=\sqrt{q^{n}}. \end{array}$$

It then follows that

$$\begin{array}{cc} \; & \left|\sum_{y\in C_{0}^{\left(e,q^{n}\right)}}\left(\sum_{f=1}^{N-1}e_{f}\sum_{x_{0}\in F_{q}^{*}}\chi \left(x_{0}y\right)\psi_{f}\left(y\right)\right)\cdots \left(\sum_{f=1}^{N-1}e_{f}\sum_{x_{k-1}\in F_{q}^{*}}\chi \left(x_{k-1}y\right)\psi_{f}\left(y\right)\right)\right|\\ = & \left|e^{-k}\left(\sum_{y\in F_{q^{n}}^{*}}\chi \left(y\right)\kappa \left(y\right)\cdots \chi \left(y\right)\kappa \left(y\right)\right)\sum_{f=1}^{N-1}e_{f}\sum_{x_{0}\in F_{q}^{*}}\sum_{\eta \in \hat{C}}{\bar{\psi }}_{f}\left(x_{0}\right)\bar{\eta }\left(x_{0}\right)\cdots \right.\\ \; & \;\;\;\;\;\;\;\;\;\;\;\;\;\;\;\;\;\;\;\;\;\;\;\;\;\;\;\;\;\;\;\;\;\;\;\;\;\;\;\;\;\;\;\;\;\;\;\;\;\;\;\;\;\;\;\;\;\;\;\;\;\;\left.\sum_{f=1}^{N-1}e_{f}\sum_{x_{k-1}\in F_{q}^{*}}\sum_{\eta \in \hat{C}}{\bar{\psi }}_{f}\left(x_{k-1}\right)\bar{\eta }\left(x_{k-1}\right)\right|\\ = & \left|e^{-k}\left(\sum_{y\in F_{q^{n}}^{*}}\chi_{k}\left(y\right)\kappa^{k}\left(y\right)\right)\sum_{f=1}^{N-1}e_{f}\sum_{x_{0}\in F_{q}^{*}}\sum_{\eta \in \hat{C}}{\bar{\psi }}_{f}\left(x_{0}\right)\bar{\eta }\left(x_{0}\right)\cdots \right.\\ & \;\;\;\;\;\;\;\;\;\;\;\;\;\;\;\;\;\;\;\;\;\;\;\;\;\;\;\;\;\;\;\;\;\;\;\;\;\;\;\;\;\;\;\left.\sum_{f=1}^{N-1}e_{f}\sum_{x_{k-1}\in F_{q}^{*}}\sum_{\eta \in \hat{C}}{\bar{\psi }}_{f}\left(x_{k-1}\right)\bar{\eta }\left(x_{k-1}\right)\right|\\ \leq & e^{-k}\sqrt{q^{n}}\sum_{f=1}^{N-1}\left|e_{f}\sum_{x_{0}\in F_{q}^{*}}\sum_{\eta \in \hat{C}}\bar{\eta }\left(x_{0}\right){\bar{\psi }}_{f}\left(x_{0}\right)\right|\cdots \sum_{f=1}^{N-1}\left|e_{f}\sum_{x_{k-1}\in F_{q}^{*}}\sum_{\eta \in \hat{C}}\bar{\eta }\left(x_{k-1}\right){\bar{\psi }}_{f}\left(x_{k-1}\right)\right|\\ = & e^{-k}\sqrt{q^{n}}\sum_{f=1}^{N-1}\left|e_{f}\sum_{t=0}^{q-2}\sum_{j=0}^{e-1}{\bar{\psi }}_{jN}\left(\alpha^{Tt}\right){\bar{\psi }}_{f}\left(\alpha^{Tt}\right)\right|\cdots \sum_{f=1}^{N-1}\left|e_{f}\sum_{t=0}^{q-2}\sum_{j=0}^{e-1}{\bar{\psi }}_{jN}\left(\alpha^{Tt}\right){\bar{\psi }}_{f}\left(\alpha^{Tt}\right)\right|\\ = & \sqrt{q^{n}}{\left(e^{-1}\sum_{f=1}^{N-1}\left|e_{f}\sum_{t=0}^{q-2}\zeta_{L}^{-fTt}\sum_{j=0}^{e-1}\zeta_{L}^{-TNjt}\right|\right)}^{k}\\ = & \sqrt{q^{n}}{\left(e^{-1}\sum_{f=1}^{N-1}\left|e_{f}\sum_{t=0}^{q-2}\zeta_{q-1}^{-ft}\sum_{j=0}^{e-1}\zeta_{e}^{-Tjt}\right|\right)}^{k}. \end{array}$$

We now consider the following two cases.

Case 1: If $\zeta_{e}^{-Tt}=1$ (i.e., t∈{0,e,2e,3e,⋯}), we have

$$\sum_{j=0}^{e-1}\zeta_{e}^{-Tjt}=e.$$

Case 2: If $\zeta_{e}^{-Tt}\neq 1$, we have

$$\sum_{j=0}^{e-1}\zeta_{e}^{-Tjt}=\frac{1-\zeta_{e}^{-Tet}}{1-\zeta_{e}^{-Tt}}=0.$$

Based on the above discussion, we arrive at the following conclusion.

$$\begin{array}{cc} \; & \left|\sum_{y\in C_{0}^{\left(e,q^{n}\right)}}\left(\sum_{f=1}^{N-1}e_{f}\sum_{x_{0}\in F_{q}}\chi \left(x_{0}y\right)\psi_{f}\left(y\right)\right)\cdots \left(\sum_{f=1}^{N-1}e_{f}\sum_{x_{k-1}\in F_{q}}\chi \left(x_{k-1}y\right)\psi_{f}\left(y\right)\right)\right|\\ \leq & \sqrt{q^{n}}{\left(e^{-1}\sum_{f=1}^{N-1}\left|e_{f}\sum_{t=0}^{q-2}\zeta_{q-1}^{-ft}\sum_{j=0}^{e-1}\zeta_{e}^{-Tjt}\right|\right)}^{k}\\ = & \sqrt{q^{n}}{\left(e\cdot e^{-1}\sum_{f=1}^{N-1}\left|e_{f}\sum_{eu=0}^{q-2}\zeta_{q-1}^{-feu}\right|\right)}^{k}\\ = & \sqrt{q^{n}}{\left(e\cdot e^{-1}\sum_{f=1}^{N-1}\left|e_{f}\sum_{u=0}^{d-1}\zeta_{d}^{-fu}\right|\right)}^{k}\;\;\;\;\;\;\;\;\;\;\;\;\;\;\;\;\;\;\;\;\;\;\;\;\;\;\;\;\;\;\;\;\;\;\;\;\;\;\;\;\;\;\;\;\;\;\;\;\;\;\;\;\;\;\;\;\;\;\;\;\;\;\;\;\;\;\;\;\;\;\;\;\;\;\\ = & \sqrt{q^{n}}{\left(d\sum_{f=1}^{T-1}\left|e_{fd}\right|\right)}^{k}\\ \leq & \sqrt{q^{n}}{\left(Md\right)}^{k}. \end{array}$$

This completes the proof. □

## 4. Bounds on the Aperiodic Hamming Correlation of Some FHSs Constructed via Trace Functions

In this section, we will apply the above bound on the hybrid incomplete exponential sum to derive the bounds on the aperiodic Hamming correlation of some FHSs presented in [13].

**Construction 1**  ([13]). *For each nonzero vector $b=\left(b_{0},b_{1},\cdots ,b_{k-1}\right)\in F_{q^{n}}^{k}$, define an FHS $u_{b}={\left\{u_{b}\left(t\right)\right\}}_{t=0}^{N-1}$ of length $N=\frac{q^{n}-1}{e}$ over $F_{q^{n}}^{k}$ as*

$$u_{b}\left(t\right)=\left({\mathrm{Tr}}_{q^{n}/q}\left(b_{0}\beta^{t}\right),{\mathrm{Tr}}_{q^{n}/q}\left(b_{1}\beta^{t}\right),\cdots ,{\mathrm{Tr}}_{q^{n}/q}\left(b_{k-1}\beta^{t}\right)\right),$$

*where 0≤t<N. And for any 0≤i≤e−1, let $b_{i}=\alpha^{i}b=\left(\alpha^{i}b_{0},\alpha^{i}b_{1},\cdots ,\alpha^{i}b_{k-1}\right)$. Define the FHS set as $U_{b}=\left\{u_{b_{i}}:0\leq i\leq e-1\right\}$, where*

$$u_{b_{i}}=\left(u_{b_{i}}\left(0\right),u_{b_{i}}\left(1\right),\cdots ,u_{b_{i}}\left(N-1\right)\right),$$

*and*

$$u_{b_{i}}\left(t\right)=\left({\mathrm{Tr}}_{q^{n}/q}\left(\alpha^{i}b_{0}\beta^{t}\right),{\mathrm{Tr}}_{q^{n}/q}\left(\alpha^{i}b_{1}\beta^{t}\right),\cdots ,{\mathrm{Tr}}_{q^{n}/q}\left(\alpha^{i}b_{k-1}\beta^{t}\right)\right).$$

Before calculating the aperiodic Hamming correlation of the FHS set in Construction 1, we first present the following lemmas.

**Lemma 4**  ([20]). *For any integer τ≥0, define*

(4) $$\begin{array}{c} \delta_{\tau }\left(t\right)=\left\{\begin{array}{cc} 1, & \mathrm{if}\;\;0\leq t\leq N-1-\tau ,\\ 0, & \mathrm{otherwise}, \end{array}\right. \end{array}$$

*and*

(5) $$\begin{array}{c} \sigma_{\tau }\left(k\right)=\sum_{t=0}^{N-1}\delta_{\tau }\left(t\right)\zeta_{N}^{kt},\;\;\;0\leq k\leq N-1. \end{array}$$

*By applying the inverse discrete Fourier transform, we have*

(6) $$\begin{array}{c} \delta_{\tau }\left(t\right)=N^{-1}\sum_{k=0}^{N-1}{\bar{\sigma }}_{\tau }\left(k\right)\zeta_{N}^{kt},\;\;\;0\leq t\leq N-1. \end{array}$$

**Lemma 5** 
([20]). *For any integer τ≥0, we have*

(7) $$\begin{array}{c} \sum_{k=1}^{T-1}\left|\sigma_{\tau }\left(kd\right)\right|\leq \sum_{k=1}^{T-1}\frac{1}{\sin\frac{k\pi }{T}}<\frac{2T}{\pi }\ln\left(\frac{4T}{\pi }\right). \end{array}$$

**Lemma 6.** 
*Let $\left(x_{0},x_{1},\cdots ,x_{k-1}\right)\in F_{q}^{k}$; for any non-trivial additive character χ of $F_{q^{n}}$, we have*

(8) $$\begin{array}{c} \sum_{y\in C_{0}^{\left(e,q^{n}\right)}}\sum_{x_{0}\in F_{q}}\chi \left(x_{0}y\right)\sum_{x_{1}\in F_{q}}\chi \left(x_{1}y\right)\cdots \sum_{x_{k-1}\in F_{q}}\chi \left(x_{k-1}y\right)=\frac{q^{n}-q^{k}}{e}. \end{array}$$

**Proof of Lemma 6.**  Assume that η is a multiplicative character of order *e* of $F_{q^{n}}$. According to Lemma 2, we have

$$\begin{array}{c} \sum_{g\in F_{q^{n}}}\chi \left(\sum_{s=0}^{k-1}x_{s}g^{e}\right)=\sum_{t=1}^{e-1}{\bar{\eta }}^{t}\left(\sum_{s=0}^{k-1}x_{s}\right)G\left(\eta^{t},\chi \right). \end{array}$$

Since χ is a non-trivial additive character of $F_{q^{n}}$ and $N=\frac{q^{n}-1}{e}$; it follows that

$$\begin{array}{cc} \; & \sum_{y\in C_{0}^{\left(e,q^{n}\right)}}\sum_{x_{0}\in F_{q}}\chi \left(x_{0}y\right)\sum_{x_{1}\in F_{q}}\chi \left(x_{1}y\right)\cdots \sum_{x_{k-1}\in F_{q}}\chi \left(x_{k-1}y\right)\\ = & N+\sum_{\left(x_{0},x_{1},\cdots ,x_{k-1}\right)\in F_{q}^{k}\setminus \left\{0_{k}\right\}}e^{-1}\left(\sum_{g\in F_{q^{n}}}\chi \left(\sum_{s=0}^{k-1}x_{s}g^{e}\right)-1\right)\\ = & N+\sum_{\left(x_{0},x_{1},\cdots ,x_{k-1}\right)\in F_{q}^{k}\setminus \left\{0_{k}\right\}}e^{-1}\left(\sum_{t=1}^{e-1}{\bar{\eta }}^{t}\left(\sum_{s=0}^{k-1}x_{s}\right)G\left(\eta^{t},\chi \right)-1\right)\\ = & N-\frac{q^{k}-1}{e}+e^{-1}\sum_{t=1}^{e-1}G\left(\eta^{t},\chi \right)\sum_{\left(x_{0},x_{1},\cdots ,x_{k-1}\right)\in F_{q}^{k}\setminus \left\{0_{k}\right\}}{\bar{\eta }}^{t}\left(\sum_{s=0}^{k-1}a_{s}x_{s}\right). \end{array}$$

In addition, since $T=\frac{q^{n}-1}{q-1}$ and e|(q−1), we have T≡n(mode). Given that gcd(e,n)=1, we conclude that gcd(T,e)=1. Thus, for any t∈{1,2,⋯,e−1}, $\eta^{t}$ is a non-trivial multiplicative character of $F_{q}$. Therefore, there exists $x\in F_{q}^{*}$ such that ${\bar{\eta }}^{t}\left(x\right)\neq 1$, it follows that

$$\begin{array}{cc} \; & {\bar{\eta }}^{t}\left(x\right)\sum_{\left(x_{0},x_{1},\cdots ,x_{k-1}\right)\in F_{q}^{k}\setminus \left\{0_{k}\right\}}{\bar{\eta }}^{t}\left(\sum_{s=0}^{k-1}x_{s}\right)\\ = & \sum_{\left(x_{0},x_{1},\cdots ,x_{k-1}\right)\in F_{q}^{k}\setminus \left\{0_{k}\right\}}{\bar{\eta }}^{t}\left(\sum_{s=0}^{k-1}xx_{s}\right)\\ = & \sum_{\left(x_{0},x_{1},\cdots ,x_{k-1}\right)\in F_{q}^{k}\setminus \left\{0_{k}\right\}}{\bar{\eta }}^{t}\left(\sum_{s=0}^{k-1}x_{s}\right). \end{array}$$

Because $\left(x_{0},x_{1},\cdots ,x_{k-1}\right)$ runs over $F_{q}^{k}\setminus \left\{0_{k}\right\}$, the vector $\left(xx_{0},xx_{1},\cdots ,xx_{k-1}\right)$ also runs over $F_{q}^{k}\setminus \left\{0_{k}\right\}$ for any $x\in F_{q}^{*}$. Thus,

$$\left({\bar{\eta }}^{t}\left(x\right)-1\right)\sum_{\left(x_{0},x_{1},\cdots ,x_{k-1}\right)\in F_{q}^{k}\setminus \left\{0_{k}\right\}}{\bar{\eta }}^{t}\left(\sum_{s=0}^{k-1}x_{s}\right)=0.$$

Since ${\bar{\eta }}^{t}\left(x\right)\neq 1$ for some $x\in F_{q}^{*}$, we obtain

$$\sum_{\left(x_{0},x_{1},\cdots ,x_{k-1}\right)\in F_{q}^{k}\setminus \left\{0_{k}\right\}}{\bar{\eta }}^{t}\left(\sum_{s=0}^{k-1}x_{s}\right)=0.$$

Hence, we have

$$\begin{array}{cc} \; & \sum_{y\in C_{0}^{\left(e,q^{n}\right)}}\sum_{x_{0}\in F_{q}}\chi \left(x_{0}y\right)\sum_{x_{1}\in F_{q}}\chi \left(x_{1}y\right)\cdots \sum_{x_{k-1}\in F_{q}}\chi \left(x_{k-1}y\right)\\ = & N-\frac{q^{k}-1}{e}+e^{-1}\sum_{t=1}^{e-1}G\left(\eta^{t},\chi \right)\sum_{\left(x_{0},x_{1},\cdots ,x_{k-1}\right)\in F_{q}^{k}\setminus \left\{0_{k}\right\}}{\bar{\eta }}^{t}\left(\sum_{s=0}^{k-1}x_{s}\right)\\ = & N-\frac{q^{k}-1}{e}+e^{-1}\sum_{t=1}^{e-1}G\left(\eta^{t},\chi \right)\cdot 0\\ = & N-\frac{q^{k}-1}{e}\\ = & \frac{q^{n}-1}{e}-\frac{q^{k}-1}{e}\\ = & \frac{q^{n}-q^{k}}{e}. \end{array}$$

This completes the proof. □

Below, we will derive the bounds on the aperiodic Hamming correlation of some FHSs in Construction 1.

**Theorem 2.** 
*Let $u_{b_{i}}$ and $u_{b_{j}}\;\left(0\leq i,j\leq e-1\right)$ be any two FHSs in the sequence set in Construction 1. Then, for i≠j or τ≠0, their non-trivial aperiodic Hamming correlation satisfies*

(9) $$\begin{array}{c} \left|A_{u_{b_{i}},u_{b_{j}}}\left(\tau \right)-{\left(\frac{N-\tau }{N}\right)}^{k}\frac{q^{n-k}-1}{e}\right|<\sqrt{q^{n}}{\left(\frac{2}{q\pi }\ln\left(\frac{4T}{\pi }\right)\right)}^{k}. \end{array}$$

**Proof of Theorem 2.**  According to the definition of the aperiodic Hamming correlation, the aperiodic Hamming correlation between FHSs $u_{b_{i}}$ and $u_{b_{j}}$ at a shift 0≤τ≤N−1 is given by

$$\begin{array}{cc} A_{u_{b_{i}},u_{b_{j}}}\left(\tau \right)\; & =|0\leq t\leq N-1-\tau :u_{b_{i}}\left(t\right)=u_{b_{j}}\left(t+\tau \right)|\\ \; & =|0\leq t\leq N-1-\tau :u_{b_{i}}\left(t\right)-u_{b_{j}}\left(t+\tau \right)=0_{k}|\\ \; & =|0\leq t\leq N-1-\tau ,0\leq s\leq k-1:{\mathrm{Tr}}_{q^{n}/q}\left(\alpha^{i}b_{s}\beta^{t}\right)={\mathrm{Tr}}_{q^{n}/q}\left(\alpha^{j}b_{s}\beta^{t+\tau }\right)|\\ \; & =|0\leq t\leq N-1-\tau ,0\leq s\leq k-1:{\mathrm{Tr}}_{q^{n}/q}\left(\beta^{t}b_{s}\left(\alpha^{i}-\alpha^{j}\beta^{\tau }\right)\right)=0|\\ \; & =|0\leq t\leq N-1-\tau ,0\leq s\leq k-1:{\mathrm{Tr}}_{q^{n}/q}\left(ab_{s}\alpha^{et}\right)=0|. \end{array}$$

Note that the equation $a=\alpha^{i}-\alpha^{j}\beta^{\tau }=0$ holds only in the trivial case where i=j and τ=0. When i≠j and τ=0, it is clear that $A_{u_{b_{i}},u_{b_{j}}}\left(\tau \right)=0$. Now we will discuss the cases where i≠j or τ≠0. Let $y=\alpha^{et}\neq 0$; by the definition of the additive character and Lemma 4, we obtain

$$\begin{array}{cc} A_{u_{b_{i}},u_{b_{j}}}\left(\tau \right)= & q^{-k}\sum_{t=0}^{N-1-\tau }\sum_{\left(x_{0},x_{1},\cdots ,x_{k-1}\right)\in F_{q}^{k}}\zeta_{p}^{{\mathrm{Tr}}_{q/p}\left(\sum_{s=0}^{k-1}x_{s}{\mathrm{Tr}}_{q^{n}/q}\left(ab_{s}\alpha^{et}\right)\right)}\;\;\;\;\;\;\;\;\;\;\;\;\;\;\;\;\;\;\;\;\;\;\;\;\;\;\;\;\;\;\;\;\;\;\;\;\;\;\;\;\;\;\;\;\;\;\;\\ = & q^{-k}\sum_{t=0}^{N-1-\tau }\sum_{\left(x_{0},x_{1},\cdots ,x_{k-1}\right)\in F_{q}^{k}}\chi \left(\sum_{s=0}^{k-1}x_{s}ab_{s}\alpha^{et}\right)\\ \;\;\;\;\;\;\;\;\;\;\;\;\;\;\;\;\;= & q^{-k}\sum_{t=0}^{N-1}\sum_{\left(x_{0},x_{1},\cdots ,x_{k-1}\right)\in F_{q}^{k}}\chi \left(\sum_{s=0}^{k-1}x_{s}ab_{s}\alpha^{et}\right)\delta_{\tau }^{k}\left(t\right)\\ = & {\left(qN\right)}^{-k}\sum_{t=0}^{N-1}\underset{k\;\;\mathrm{items}}{\underset{︸}{\sum_{f=0}^{N-1}\cdots \sum_{f=0}^{N-1}}}\sum_{\left(x_{0},x_{1},\cdots ,x_{k-1}\right)\in F_{q}^{k}}\chi \left(\sum_{s=0}^{k-1}x_{s}ab_{s}\alpha^{et}\right){\bar{\sigma }}_{\tau }^{k}\left(f\right){\left(\zeta_{N}^{ft}\right)}^{k}\\ = & {\left(qN\right)}^{-k}\sum_{y\in C_{0}^{\left(e,q^{n}\right)}}\left(\sum_{f=0}^{N-1}\sum_{x_{0}\in F_{q}}\chi \left(x_{0}ab_{0}y\right){\bar{\sigma }}_{\tau }\left(f\right)\zeta_{N}^{ft}\right)\cdots \\ \; & \;\;\;\;\;\;\;\;\;\;\;\;\;\;\;\;\;\;\;\;\;\;\;\;\;\;\;\;\;\;\;\;\;\;\;\;\;\;\;\;\;\;\;\;\;\;\;\;\;\left(\sum_{f=0}^{N-1}\sum_{x_{k-1}\in F_{q}}\chi \left(x_{k-1}ab_{k-1}y\right){\bar{\sigma }}_{\tau }\left(f\right)\zeta_{N}^{ft}\right). \end{array}$$

For any 0≤s≤k−1, let $\chi_{ab_{s}}$ be a non-trivial additive character of $F_{q^{n}}$. Below, we proceed to discuss the following three cases.

Case 1: When f=0, by Lemmas 4 and 6, we have

$$\begin{array}{cc} \; & \sum_{y\in C_{0}^{\left(e,q^{n}\right)}}\left(\sum_{x_{0}\in F_{q}}\chi \left(x_{0}ab_{0}y\right){\bar{\sigma }}_{\tau }\left(0\right)\zeta_{N}^{0}\right)\cdots \left(\sum_{x_{k-1}\in F_{q}}\chi \left(x_{k-1}ab_{k-1}y\right){\bar{\sigma }}_{\tau }\left(0\right)\zeta_{N}^{0}\right)\\ = & {\left(N-\tau \right)}^{k}\sum_{y\in C_{0}^{\left(e,q^{n}\right)}}\left(\sum_{x_{0}\in F_{q}}\chi_{ab_{0}}\left(x_{0}y\right)\right)\cdots \left(\sum_{x_{k-1}\in F_{q}}\chi_{ab_{k-1}}\left(x_{k-1}y\right)\right)\\ = & {\left(N-\tau \right)}^{k}\left(\frac{q^{n}-q^{k}}{e}\right). \end{array}$$

Case 2: When f>0 and $\left(x_{0},x_{1},\cdots ,x_{k-1}\right)=0_{k}$, we have

$$\begin{array}{cc} \; & \sum_{y\in C_{0}^{\left(e,q^{n}\right)}}\left(\sum_{f=1}^{N-1}\chi \left(0\right){\bar{\sigma }}_{\tau }\left(f\right)\zeta_{N}^{ft}\right)\cdots \left(\sum_{f=1}^{N-1}\chi \left(0\right){\bar{\sigma }}_{\tau }\left(f\right)\zeta_{N}^{ft}\right)\\ = & \sum_{t=0}^{N-1}\left(\sum_{f=1}^{N-1}{\bar{\sigma }}_{\tau }\left(f\right)\zeta_{N}^{ft}\right)\cdots \left(\sum_{f=1}^{N-1}{\bar{\sigma }}_{\tau }\left(f\right)\zeta_{N}^{ft}\right)\\ = & \sum_{f=1}^{N-1}{\bar{\sigma }}_{\tau }\left(f\right)\cdots \sum_{f=1}^{N-1}{\bar{\sigma }}_{\tau }\left(f\right)\sum_{t=0}^{N-1}{\left(\zeta_{N}^{ft}\right)}^{k}\\ = & \sum_{f=1}^{N-1}{\bar{\sigma }}_{\tau }\left(f\right)\cdots \sum_{f=1}^{N-1}{\bar{\sigma }}_{\tau }\left(f\right)\left(\frac{1-\zeta_{N}^{Nfk}}{1-\zeta_{N}^{fk}}\right)\\ = & \sum_{f=1}^{N-1}{\bar{\sigma }}_{\tau }\left(f\right)\cdots \sum_{f=1}^{N-1}{\bar{\sigma }}_{\tau }\left(f\right)\cdot 0\\ = & 0. \end{array}$$

Case 3: When f>0 and $\left(x_{0},x_{1},\cdots ,x_{k-1}\right)\neq 0_{k}$, by the definition of the multiplicative character, Theorem 1, and Lemma 5, we have

$$\begin{array}{cc} \; & \left|\sum_{y\in C_{0}^{\left(e,q^{n}\right)}}\left(\sum_{f=1}^{N-1}\sum_{x_{0}\in F_{q}^{*}}\chi \left(x_{0}ab_{0}y\right){\bar{\sigma }}_{\tau }\left(f\right)\zeta_{N}^{ft}\right)\cdots \left(\sum_{f=1}^{N-1}\sum_{x_{k-1}\in F_{q}^{*}}\chi \left(x_{k-1}ab_{k-1}y\right){\bar{\sigma }}_{\tau }\left(f\right)\zeta_{N}^{ft}\right)\right|\\ = & \left|\sum_{y\in C_{0}^{\left(e,q^{n}\right)}}\left(\sum_{f=1}^{N-1}\sum_{x_{0}\in F_{q}^{*}}\chi_{ab_{0}}\left(x_{0}y\right){\bar{\sigma }}_{\tau }\left(f\right)\psi_{f}\left(y\right)\right)\cdots \left(\sum_{f=1}^{N-1}\sum_{x_{k-1}\in F_{q}^{*}}\chi_{ab_{k-1}}\left(x_{k-1}y\right){\bar{\sigma }}_{\tau }\left(f\right)\psi_{f}\left(y\right)\right)\right|\\ = & \left|\sum_{y\in C_{0}^{\left(e,q^{n}\right)}}\left(\sum_{f=1}^{N-1}{\bar{\sigma }}_{\tau }\left(f\right)\sum_{x_{0}\in F_{q}^{*}}\chi_{ab_{0}}\left(x_{0}y\right)\psi_{f}\left(y\right)\right)\cdots \left(\sum_{f=1}^{N-1}{\bar{\sigma }}_{\tau }\left(f\right)\sum_{x_{k-1}\in F_{q}^{*}}\chi_{ab_{k-1}}\left(x_{k-1}y\right)\psi_{f}\left(y\right)\right)\right|\\ < & \sqrt{q^{n}}{\left(\frac{\left(q-1\right)}{e}\frac{2T}{\pi }\ln\left(\frac{4T}{\pi }\right)\right)}^{k}. \end{array}$$

In summary, we conclude that

$$\begin{array}{cc} \; & \frac{1}{{\left(qN\right)}^{k}}\left[{\left(N-\tau \right)}^{k}\frac{q^{n}-q^{k}}{e}-\sqrt{q^{n}}{\left(\frac{2T(q-1)}{e\pi }\ln\left(\frac{4T}{\pi }\right)\right)}^{k}\right]<A_{u_{b_{i}},u_{b_{j}}}\left(\tau \right)<\\ \; & \;\;\;\;\;\;\;\;\;\;\;\;\;\;\;\;\;\;\;\;\;\;\;\;\;\;\;\;\;\;\;\;\;\;\;\;\;\;\;\;\;\;\frac{1}{{\left(qN\right)}^{k}}\left[{\left(N-\tau \right)}^{k}\frac{q^{n}-q^{k}}{e}+\sqrt{q^{n}}{\left(\frac{2T(q-1)}{e\pi }\ln\left(\frac{4T}{\pi }\right)\right)}^{k}\right]. \end{array}$$

Simplifying the above formula yields

$$\begin{array}{cc} \; & {\left(\frac{N-\tau }{N}\right)}^{k}\frac{q^{n-k}-1}{e}-\sqrt{q^{n}}{\left(\frac{2}{q\pi }\ln\left(\frac{4T}{\pi }\right)\right)}^{k}<A_{u_{b_{i}},u_{b_{j}}}\left(\tau \right)<\\ \; & \;\;\;\;\;\;\;\;\;\;\;\;\;\;\;\;\;\;\;\;\;\;\;\;\;\;\;\;\;\;\;\;\;\;\;\;\;\;\;\;\;\;\;\;\;\;\;\;\;\;\;\;\;\;\;\;\;{\left(\frac{N-\tau }{N}\right)}^{k}\frac{q^{n-k}-1}{e}+\sqrt{q^{n}}{\left(\frac{2}{q\pi }\ln\left(\frac{4T}{\pi }\right)\right)}^{k}. \end{array}$$

Clearly, it can be rewritten as

$$\begin{array}{c} \left|A_{u_{b_{i}},u_{b_{j}}}\left(\tau \right)-{\left(\frac{N-\tau }{N}\right)}^{k}\frac{q^{n-k}-1}{e}\right|<\sqrt{q^{n}}{\left(\frac{2}{q\pi }\ln\left(\frac{4T}{\pi }\right)\right)}^{k}, \end{array}$$

where $N=\frac{q^{n}-1}{e}$ and $T=\frac{q^{n}-1}{q-1}$. This completes the proof. □

**Remark 1.** 
*When k=1, the bound on the aperiodic Hamming correlation of FHSs in [20] can be viewed as a special case of the above bound.*

## 5. Discussion on the Bound

In this section, we will discuss the rationality of the bound on the aperiodic Hamming correlation of some FHSs as stated in Theorem 2. Before proceeding with the discussion, we first present the following lemma.

**Lemma 7** 
([20,27]). *If a positive integer e satisfies e|(q−1) and gcd(e,n)=1, then the system of linear equations*

$$\begin{array}{c} \left\{\begin{array}{cc} {\mathrm{Tr}}_{q^{n}/q}\left(ab_{0}x\right) & =0,\\ {\mathrm{Tr}}_{q^{n}/q}\left(ab_{1}x\right) & =0,\\ \; & \vdots \\ {\mathrm{Tr}}_{q^{n}/q}\left(ab_{k-1}x\right) & =0, \end{array}\right. \end{array}$$

*has exactly $\frac{q^{n-k}-1}{e}$ solutions in any coset $C_{i}^{\left(e,q^{n}\right)}$, where 0≤i≤e−1.*

According to Lemma 7, when i≠j or τ≠0, the periodic Hamming correlation between any two FHSs $u_{b_{i}}$ and $u_{b_{j}}$ in the sequence set $U_{b}$ in Construction 1 is $\frac{q^{n-k}-1}{e}$, which represents the total number of frequency collisions between two sequences $u_{b_{i}}$ and $u_{b_{j}}$ over the full period at shifts 1≤τ≤N−1. Therefore, the average number of frequency collisions between $u_{b_{i}}$ and $u_{b_{j}}$ over a correlation window of length N−τ is given by

(10) $$\begin{array}{c} {\left(\frac{N-\tau }{N}\right)}^{k}\frac{q^{n-k}-1}{e}. \end{array}$$

This average number of frequency collisions can be regarded as an estimate of the actual number of frequency collisions between sequences $u_{b_{i}}$ and $u_{b_{j}}$ over a correlation window of length N−τ. The maximum estimation error is given by

$$\begin{array}{c} \Delta =\max\left\{\left|A_{u_{b_{i}},u_{b_{j}}}\left(\tau \right)-{\left(\frac{N-\tau }{N}\right)}^{k}\frac{q^{n-k}-1}{e}\right|:i\neq j\;\;\mathrm{or}\;\;\tau \neq 0\right\}. \end{array}$$

According to Equation (9), the upper bound of Δ is

$$\begin{array}{c} Y=\sqrt{q^{n}}{\left(\frac{2}{q\pi }\ln\left(\frac{4T}{\pi }\right)\right)}^{k}. \end{array}$$

In the following example, we compute the actual maximum estimation error and the estimation error given by the theoretical bound, in order to illustrate that the average number of frequency collisions given by Equation (10) may be a useful estimate for the aperiodic Hamming correlation of some FHSs.

**Example 1.** 
*Let n=3, e=2, k=1, q=11, and $b=b_{0}=1$. Let α be a generator of $F_{11^{3}}$ defined by $\alpha^{3}+2\alpha +9=0$. Then, the FHS set $U_{b}$ in Construction 1 is composed of the following two sequences of length 665:*

$$\begin{array}{cc} \; & 3,7,8,7,1,0,2,7,8,3,6,7,4,2,4,3,2,7,9,10,7,1,8,3,\cdots \\ \; & 0,6,2,1,1,0,0,4,6,4,9,5,4,0,4,0,6,3,8,2,5,4,5,6,\cdots \end{array}$$

*On the one hand, by calculating the actual maximum estimation error, we obtain that Δ≈3.99, which was obtained through computer experiments. On the other hand, by substituting the parameters into *Υ*, we have*

$$\begin{array}{c} Y=\sqrt{q^{n}}{\left(\frac{2}{q\pi }\ln\left(\frac{4T}{\pi }\right)\right)}^{k}=\sqrt{11^{3}}\frac{2}{11\pi }\ln\left(\frac{4(11^{3}-1)}{10\pi }\right)\approx 10.84. \end{array}$$

*It can be verified that the actual maximum estimation error is smaller than the estimation error given by the bound under these parameters.*

**Figure 1:**
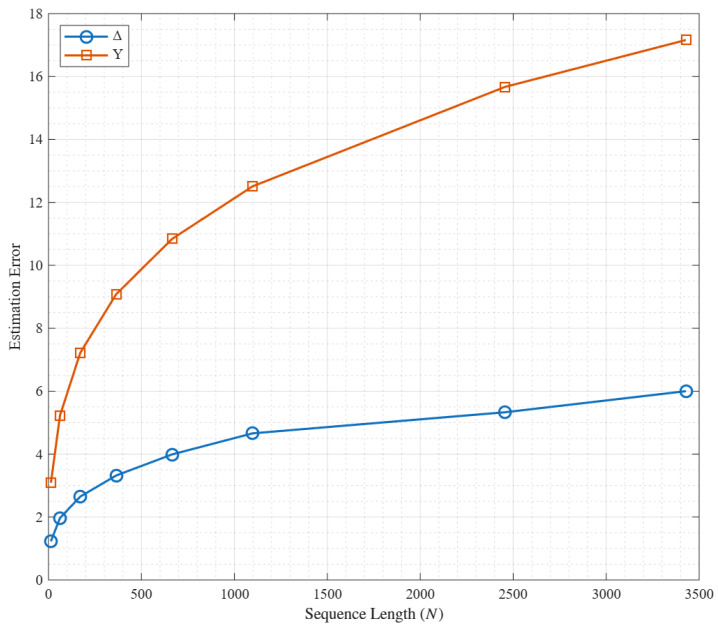
The difference between Δ and Y for n=3,e=2,k=1, and q≤19.

Now, we compare the values of Δ and Y in Table 1, where n=3,e=2,k=1, and q≤19. To further intuitively demonstrate the difference between the actual and theoretical errors, Figure 1 presents a visualization of the variations of Δ and Y under the aforementioned parameters. It can be observed that for small values of *n*, the actual maximum estimation error is much smaller than the estimation error given by the theoretical bound. It implies that the average number of frequency collisions provided by Equation (10) may be a useful estimate for the aperiodic Hamming correlation of some FHSs based on the trace functions defined before.

## 6. Conclusions

In this paper, we derive an upper bound for a hybrid incomplete exponential sum over finite fields. By applying the above bound, we derive a bound on the aperiodic Hamming correlation of some FHSs from trace functions and explicitly point out that the bound given in [20] is a special case of our result. Finally, we analyze the maximum estimation error between the average frequency collision number and the actual frequency collision number for these FHSs and illustrate the rationality of the bound on the aperiodic Hamming correlation derived herein. Determining the bounds on the aperiodic Hamming correlation of other known FHSs is both intriguing and challenging. Furthermore, the development of novel approaches to achieve tighter bounds remains an open and demanding problem. We cordially invite researchers to address these issues.

## Figures and Tables

**Table 1:** Comparison of Δ and Y for n=3,e=2,k=1, and q≤19.

*q*	*N*	Δ	Y
3	13	1.23	3.09
5	62	1.97	5.23
7	171	2.65	7.22
$3^{2}$	364	3.32	9.08
11	665	3.99	10.84
13	1098	4.66	12.51
17	2456	5.33	15.67
19	3429	6.00	17.16

## Data Availability

Data are contained within the article.
